# The antioxidant effects of riluzole on the APRE-19 celll model injury-induced by t-BHP

**DOI:** 10.1186/s12886-017-0614-0

**Published:** 2017-11-23

**Authors:** Chaolan Shen, Wei Ma, Wenbin Zheng, Hao Huang, Renchun Xia, Chu Li, Xiaobo Zhu

**Affiliations:** 1 The State Key Laboratory of Ophthalmology, Zhongshan Ophthalmic Center, Sun Yat-sen University, Guangzhou, China; 2 People’s Hospital of Guangxi Zhuang Autonomous Region, Nanning, Guangxi China; 3 Frist Affiliated Hospital of Gannan Medical University, Ganzhou, Jiangxi China; 4 People’s hospital of Deyang city, Deyang, Sichuan China

**Keywords:** Riluzole, TRAAK K_2P_ channel, ARPE-19 cells, AMD, apoptosis

## Abstract

**Background:**

Age-related macular degeneration (AMD) causes the dysfunction of the retinal pigment epithelial (RPE) cells. In this study, we examined the effects of riluzole, a sustained activator of the TRAAK potassium channel, on human RPE (ARPE-19) cells in an oxidant-induced cell-injury model and elucidate the mechanism of riluzole on RPE cell apoptosis.

**Methods:**

The follow four groups of ARPE-19 cells were treated with riluzole and/or tert-butyl hydroperoxide (t-BHP) for 24.0 h: control, t-BHP, riluzole, and t-BHP + riluzole. Cell apoptosis was measured by flow cytometry, and Western blotting was performed to analyze the expression of the weakly inward rectifying potassium (TRAAK) channel. Finally, the mitochondrial membrane potential (*Δψm*) was detected by flow cytometry, and cytochrome C (Cyt-c) release was assessed by Western blotting.

**Results:**

The viability of the cells in the cotreated group was significantly higher (85.6 ± 3.1%) than that in the t-BHP group (66.2 ± 2.5%). In addition, the cells in the cotreated group had a higher effect on increasing the expression of TRAAK than the t-BHP group. The results also showed that Cyt-c translocation significantly decreased and *Δψm* increased in the cotreated group.

**Conclusions:**

These results demonstrate that riluzole protects RPE cells from apoptosis. The protection mechanism of riluzole could be from stabilizing mitochondrial *Δψm* and preventing the release of Cyt-c. Changes in TRAAK expression might also contribute to the protection of RPE cells.

## Background

Age-related macular degeneration (AMD), the loss of macular function from degenerative cellular changes caused by aging, is the leading cause of central vision loss in the elderly in developed countries [1]. Retinal pigment epithelial (RPE) cells are thought to be involved in several degenerative diseases of the retina, including AMD. In spite of the multiple pathways that contribute to RPE cell death, caspase signaling was demonstrated to be a common pathway in RPE cell apoptosis during AMD; however, caspase inhibitors only slow down apoptosis rather than inhibit it, which suggests that rescuing cells from death during the earlier stages of apoptosis might be more efficient [2, 3].

Recent studies have focused on the weakly inward rectifying potassium channel (TRAAK), a member of the two-pore domain potassium channel (K_2P_), which is involved in degenerative diseases of the central nervous system (CNS). Expressed predominantly in CNS, K_2P_ has various important functions, such as transmitting synapses, maintaining the resting membrane potential, and regulating cell volume, as well as regulating in pathological processes [4]. The activity of the TRAAK K_2P_ channel is affected by many physiological conditions, including the presence of polyunsaturated fatty acids, levels of extracellular pH, mechanical stress, and osmotic pressure [5, 6]. Opening the TRAAK channels down regulates neuron excitability and decreases the cellular metabolic rate, which plays a key role in ischemia-induced neuronal degenerative processes [7–9].

The retina is regarded as a direct extension of the CNS, and its mechanisms for degeneration and performance have many properties similar to those of CNS. Thus, it has been proposed that TRAAK K_2P_ also plays a role in inhibiting retinal cell apoptosis, which makes TRAAK K_2P_ a new target for treating retinal degenerative disease. It is known that 2-amino-6-trifluoromethoxy benzothiazole (riluzole) is a neuroprotective drug that activates TREK/TRAAK channels. In our previous studies, we observed that riluzole could protect hRPE cells against oxidative injury–induced cell death [10], but additional studies are needed to determine the mechanisms for this effect. In this study, we elucidate the mechanism of activated TRAAK channels on human RPE (ARPE-19) cells in an oxidant-induced cell injury model.

## Methods

### Cell culture and treatment

ARPE-19 cells were obtained from American Type Culture Collection (Manassas, VA, USA). This study was approved by the Ethics Committee of the Zhongshan Ophthalmic Center and followed the tenets of the Declaration of Helsinki. These cells were cultured in Dulbecco’s Modified Eagle’s Medium/F12 (DMEM/F12; Invitrogen, Paisley, UK) plus 10.0% fetal bovine serum (Sijiqing, HZ, China) in a humidified incubator at 37.0 °C and 5.0% CO^2^. The cells were grown to an appropriate density and used at 10~15 passages.

The ARPE-19 cells were divided into four groups as follows: control, 10.0-μM riluzole (Tocris, Minneapolis, MN, USA), 300.0-μM tert-butyl hydroperoxide (t-BHP; Sigma-Aldrich, St. Louis, MO, USA), and 300.0-μM t-BHP cotreated with 10.0 μM riluzole. After reaching 80.0% confluence, the cells were incubated under different conditions for 24.0 h.

### Evaluation of apoptosis rates

**Fig. 1:**
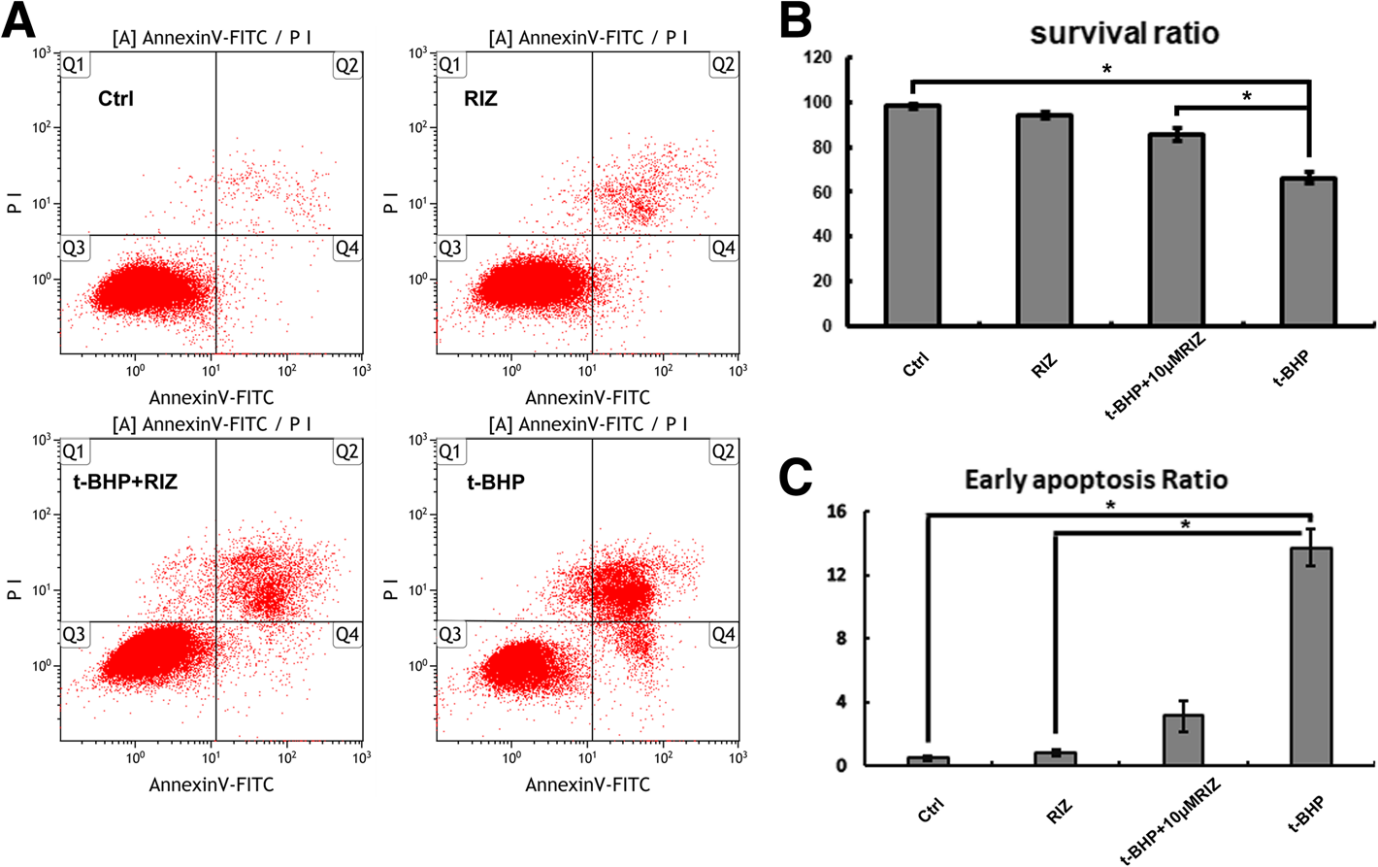
Activation of two-pore domain potassium channels (K2P) improved human retinal pigment epithelia (ARPE-19)-19 cell survival. **a** Representative annexin V/PI staining of apoptosis cells in riluzole and/or t-BHP treat with ARPE-19 cells for 24.0 h. Graphic representation of the survival ratios for ARPE-19. *P < .05 vs. t-BHP group. **b** Quantitative analysis of the survival ratio of ARPE-19 at 24.0 h post-treatment. **c** Quantitative analysis of the early apoptosis ratio of ARPE-19 at 24.0 h post-treatment. *P < .05 vs. t-BHP group

The apoptotic rates were analyzed using Flow-Count Fluorospheres (Beckman Coulter, Brea, CA, USA) and an annexin V-fluorescein isothiocyanate (FITC)/propidium iodide (PI) kit (Roche, Penzberg, Germany) in which annexin V binds to the exposed phosphatidylserine on the plasma membrane of apoptotic cells. The cells were stained according to the manufacturer’s instructions. The percentage of early apoptosis was calculated from the proportion of cells that were annexin V–positive and PI-negative (Q4 in Fig. 1a), while the percentage of late apoptosis plus necrosis was calculated from the proportion of cells that were annexin-V and PI double positive (Q2 in Fig. 1a).

### Quantitative analysis of TRAAK expression by western blotting

To assess TRAAK expression, ARPE-19 cells were homogenized in 100.0 μL lysis buffer containing 50.0 mM Tris–HCl (pH 7.5), 150.0 mM NaCl, 0.5% Triton X-100, and 1× protease inhibitor cocktail (Sigma-Aldrich, St. Louis, MO, USA). The samples were centrifuged at 11000 g at 4.0 °C for 30.0 min, after which the supernatants were collected and mixed with 5× sample loading buffer to prepare the samples.

Briefly, the protein samples were separated by sodium dodecyl sulfate polyacrylamide gel electrophoresis (SDS-PAGE) and subsequently transferred to a polyvinylidene fluoride membrane (Roche, Penzberg, Germany). The membranes were blocked in 5.0% bovine serum albumin (BSA) for 1.0 h at room temperature (RT) and incubated at 4.0 °C overnight with rabbit polyclonal anti-TRAAK antibody (1:1000; APC-108, Alomone Labs, Jerusalem, Israel). The membranes were then incubated with a horseradish peroxidase (HRP)-conjugated secondary antibody for 1.0 h at RT. The integrated optical density of each protein was normalized to that of β-actin for quantitative comparison.

### Detection of cytochrome c release

To verify the cytochrome C (Cyt-c) release, subcellular fractionations were used to detect the Cyt-c in the cytosol and mitochondria by Western blotting [11]. The cells were suspended with buffer A (20.0 mM HEPES-KOH [pH 7.5], 10.0 mM KCl, 1.5 mM MgCl2, 1.0 mM EDTA, 1.0 mM EGTA, 1.0 mM DTT, 250.0 mM sucrose, and 1× protease inhibitor cocktail) (Sigma-Aldrich, St. Louis, MO, USA) and homogenized using a Dounce homogenizer. Cell debris and nuclei were removed by centrifugation at 1000 g for 10.0 min at 4.0 °C. The supernatant was further centrifuged at 10000 g for 20.0 min, and then transferred to clean tubes and centrifuged at 100000 g for 1.0 h at 4.0 °C. The supernatant was collected as the cytosolic fraction and the pellets were collected as the mitochondrial fraction, which was resuspended in buffer A containing 0.5% (*v*/v) NP40. The cytosolic and mitochondrial fractions were mixed with 5× sample loading buffer to prepare the samples. The Western blotting protocol was followed as above with incubation of mouse monoclonal anti-Cyt-c antibody (1:1000; Cell Signaling Technology, Danvers, MA, USA). Anti β-actin (1:2000; Cell Signaling Technology, Danvers, MA, USA) and anti-COXIV antibody (1:1000, Molecular Probes, Rockford, IL, USA) were used as loading controls.

### Measurement of Δψ_m_ by JC-1

A JC-1 fluorescent probe (Merck, Darmstadt, Germany) was used to measure *Δψ*_*m*_ of ARPE-19. HRPEs were incubated with 1.0 μg/mL JC-1 for 15.0 min at 37.0 °C, centrifuged at 800 g for 5.0 min, washed with PBS, and analyzed immediately using a Beckman Gallios Flow Cytometer (Beckman Coulter, Brea, CA, USA). The photomultiplier settings were adjusted to detect green fluorescence (uEM = 525.0 nm) of the JC-1 monomers on filter 1, and red fluorescence (uEM = 590.0 nm) of the JC-1 J-aggregates on filter 2. The ratio of red to green (aggregate to monomer) fluorescence intensity was used to assess the ratio of healthy cells to apoptotic cells with low *Δψ*_*m*_.

### Statistical analyses

The data are expressed as the mean ± SD. Differences among the groups were assessed by one-way analysis of variance and the Bonferroni multiple comparison test, and all data were analyzed using SPSS 13.0 (SPSS Inc., Chicago, IL, USA). *P* < .05 was considered statistically significant.

## Results

### Activation of K_2P_ improved ARPE-19 cell survival

Whether K_2P_ improved ARPE-19 cells survival should be confirmed before investigating the role of K_2P_ in ARPE-19 cells. To address this, we measured the survival ratio in ARPE-19 cells treated with t-BHP plus riluzole or t-BHP alone using annexin V and PI staining and flow cytometry. The data showed that the survival percentage of ARPE-19 significantly decreased in the t-BHP group (66.2 ± 2.5%) over that in the control group (98.1 ± 1.0%, *P* = .004, *n* = 3) (Figs. 1a, b); however, the percentage of surviving cells significantly increased in the t-BHP + riluzole group (85.6 ± 3.1%, *P* = .011, n = 3) compared with that in the t-BHP alone group (Figs. 1a, b). Furthermore, we detected the cells’ early apoptosis after t-BHP and/or riluzole treatment. The data showed that early apoptosis significantly decreased in the t-BHP + riluzole group (3.2 ± 1.7%) compared to that in the t-BHP group (13.7 ± 2.0%, *P* = .001, n = 3) (Figs. 1a, c).

### Quantitative expression of TRAAK K_2P_ in ARPE-19 cells

**Fig. 2:**
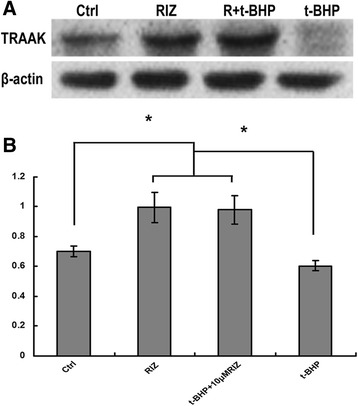
Riluzole increased TRAAK expression levels in human retinal pigment epithelia (ARPE-19) cells. **a** The expression of TRAAK was determined by Western blotting. *β*-actin was used as an internal control. **b** Quantitative analysis of the protein level of TRAAK in ARPE-19 cells. The data were presented as the mean ± SD relative to the control group. *P < .05

To investigate the quantitative expression of TRAAK, we examined the TRAAK protein expression in ARPE-19 cells using Western blotting. When exposing the cells to riluzole for 24.0 h, the normalized expression of TRAAK in riluzole in the single and cotreated groups was 1.0 ± 0.1 and 0.98 ± 0.1, respectively. In contrast, the expression in the vehicle and t-BHP groups decreased to 0.70 ± 0.04 and 0.61 ± 0.04, respectively. The data showed that riluzole can dramatically increase TRAAK expression (*P* = .001, *n* = 4) (Figs. 2a, b).

### Activation of K_2P_ prevents cytochrome c release in cultured ARPE-19 cells

**Fig. 3:**
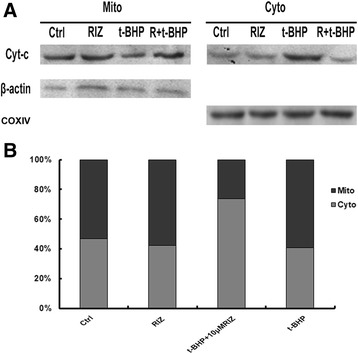
Activation of two-pore domain potassium channels (K2P) prevents cytochrome c (Cyt-c) release to the cytoplasm in culture human retinal pigment epithelia (ARPE-19) cells. **a** The release of mitochondrial Cyt-c was estimated by examining the Cyt-c protein content in mitochondria and the extracted mitochondria-free cytosolic fraction by Western blot. COXIV and β-actin were used as loading controls. **b** Quantitative analysis of the percentage of Cyt-c in mitochondria and cytoplasm of ARPE-19 cells

As is known, Cyt-c is an apoptotic factor normally located in the mitochondria. It is also known that activation of caspases and initiation of apoptosis will stimulate Cyt-c leakage from the mitochondria into the cytoplasm [12]; therefore, we detected Cyt-c by Western blot analysis. The data showed that more Cyt-c was released from the mitochondria into the cytoplasm in the t-BHP treated ARPE-19 cells than in the vehicle cells. Moreover, the ratio of Cyt-c in mitochondria and cytoplasm dropped from 0.97 ± 0.05 (control group) to 0.36 ± 0.01 in the t-BHP group (*P* < .05, *n* = 3); however, Cyt-c translocation decreased in the riluzole + t-BHP and riluzole groups compared with that in the t-BHP group (Figs. 3a, b). The ratio significantly increased in the riluzole (1.21 ± 0.03) and riluzole + t-BHP groups (1.06 ± 0.05), respectively. These data indicated that riluzole protects RPE cells by inhibiting Cyt-c translocation from the mitochondria into the cytoplasm.

### Activation of K_2P_ prevents the loss of mitochondrial Δψm

**Fig. 4:**
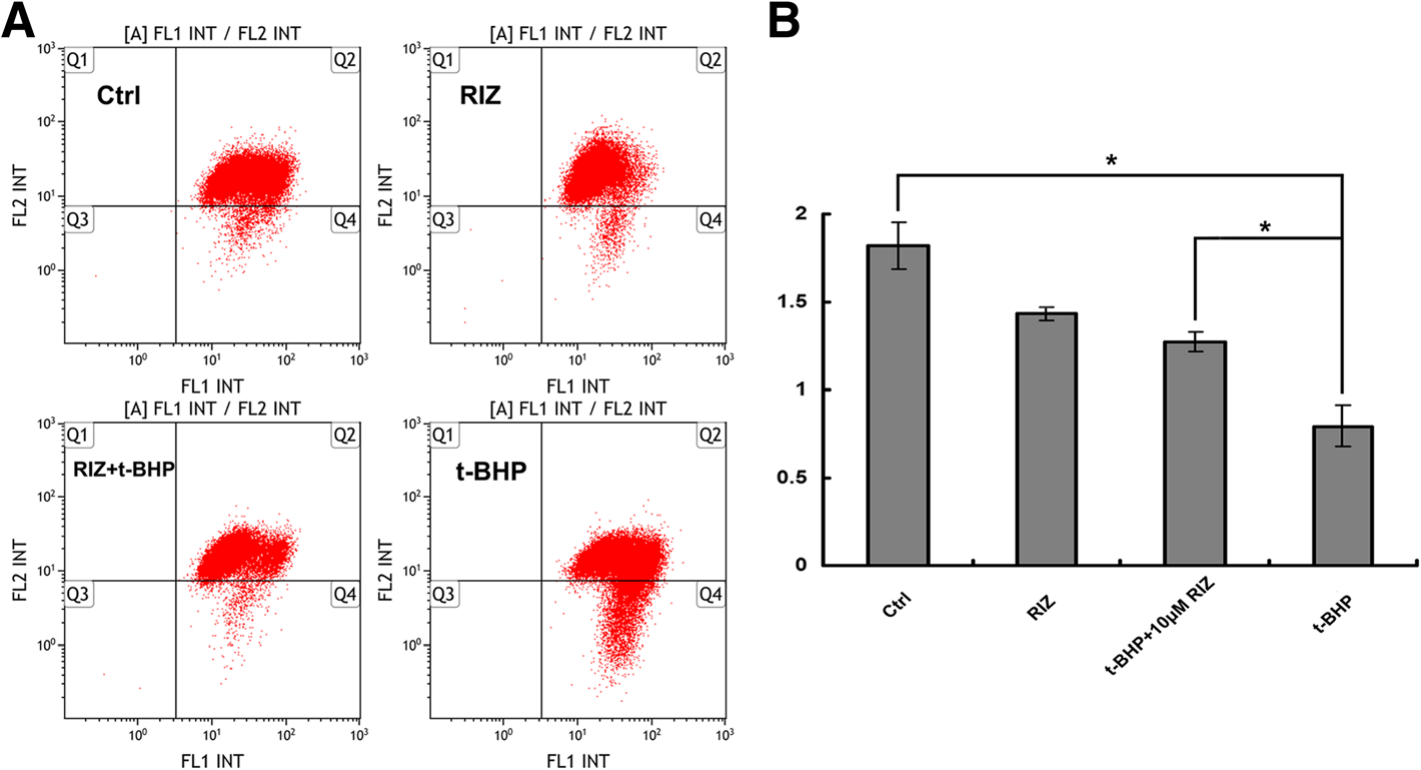
Activation of two-pore domain potassium channels (K2P) prevented the loss of mitochondrial membrane potential (Δψm). **a** *Δψ*_*m*_ of human retinal pigment epithelia (ARPE-19) cells was measured by flow cytometry after JC-1 staining. t-BHP treatment resulted in a rapid loss of *Δψ*_*m*_ in ARPE-19; however, the addition of riluzole attenuated that loss. **b** Quantitative analysis of *Δψ*_*m*_ in ARPE-19 cells. Values represent the mean ± SD of each separate experiment performed in triplicate. *P < 0.05 vs. the t-BHP group

To determine whether the mitochondria were involved in the protective effects of activated K_2P_ on apoptosis, *Δψ*_*m*_ was measured in ARPE-19 cells after treating with riluzole and/or t-BHP for 24.0 h. We found that *Δψ*_*m*_ significantly decreased in the t-BHP group (0.79 ± 0.04) compared with that in the vehicle group (1.82 ± 0.13, *P* = .001, *n* = 3); however, *Δψ*_*m*_ significantly increased in the ARPE-19 cells in the riluzole + t-BHP group (1.27 ± 0.06) over that in the t-BHP group (*P* = .002, n = 3) (Figs. 4a, b). These data suggested that riluzole-activated K_2P_ prevents *Δψ*_*m*_ loss during apoptosis caused by oxidative stress.

## Discussion

RPE cells constitute a simple layer of cuboidal cells that are localized behind the photoreceptor (PR) cells. The main sites of light energy absorption are the melanin pigment granules in the RPE cells, which are most susceptible to oxidative stress in many primary and secondary AMDs [13, 14]. Injury to RPE cells from oxidative stress is considered to be one of the crucial contributors to the pathogenesis of AMD. Associations might exist among the RPE cells, retinal neurons, and TRAAK channel neuroprotection. Riluzole is a neuroprotective drug that activates TREK/TRAAK channels and is licensed for the treatment of amyotrophic lateral sclerosis and in clinical trial for spinal cord injuries [7, 8, 15]. Thus far, research on the neuroprotective effects of riluzole is ongoing; however, to our knowledge, no study on riluzole in degenerative retinopathy has been reported. In our previous study, we observed TRAAK channel protein expression in human RPE cells, which might have relevance in cell protection. Further, we selected riluzole as an activator for TRAAK K_2P_ to study the protective mechanism of TRAAK K_2_p during oxidative injury.

Because it is difficult to obtain fresh human eyeballs, we switched our investigation to ARPE-19 cells as an alternative choice in this study. We demonstrated that riluzole-activated K_2P_ significantly decreased early apoptosis induced by t-BHP (Fig. 1a) in ARPE-19 cells. This suggested that the TRAAK K_2P_ channels participate in the protective effects on ARPE-19 cells at the early stage of apoptosis. In addition, we demonstrated that riluzole has the effect of maintaining normal cell structure during oxidative injury [10]. Previous researchers have found that the riluzole-activated TRAAK channel opposes membrane depolarization and cell excitability [16, 17]. Thus, we proposed that the protective role of riluzole against apoptosis is a result of its capability to activate TRAAK-current potency.

To evaluate the protective effects of activated K_2P_ on mitochondrial function, *Δψm* and the release of Cyt-c from the mitochondria into the cytoplasm were measured. Our data showed a rapid loss of *Δψ*_*m*_ and a marked release of Cyt-c under oxidative injury conditions; however, the addition of riluzole prevented *Δψ*_*m*_ loss and the release of Cyt-c. The loss of *Δψ*_*m*_ is an initial event during oxidative stress and indicates dysfunction of the mitochondria [18, 19]. The release of Cyt-c implies a disruption of the outer mitochondrial membrane. Our study suggests that activation of K_2P_ can attenuate the damage to mitochondrial function under oxidative stress conditions.

Another interesting discovery from this study was the upregulation of TRAAK in RPE cells when cotreated with riluzole. Using Western blotting, the mean expression of TRAAK markedly increased in the riluzole groups (Fig. 2). t-BHP treatment increases ARPE-19 apoptosis and decreases TRAAK expression, while riluzole protects ARPE-19 cells against t-BHP-induced injury and increases TRAAK expression. Activation of the TRAAK channel during the early periods of apoptosis will also oppose depolarization of the cell membrane and reduce Ca^2+^ influx to help protect the cells from further damage [20]. Xu et al. [21] found that TRAAK mRNA increased by 70.0% in the cortex after ischemia. Based on our current results, it is most likely that the changes in TRAAK expression are associated with apoptosis. Whether the quantity changes of TRAAK contribute to RPE cell protection is not fully understood. Further studies are required to find the exact mechanisms and interactions of the TRAAK proteins essential for the retina protection.

## Conclusions

Our results demonstrate that the activation of K_2P_ induced by riluzole attenuates t-BHP-induced injury to ARPE-19 cells, which contributes to stabilizing *Δψ*_*m*_ and preventing the release of Cyt-c from the mitochondria. The changes in TRAAK expression might also have some relevance in cell protection. TRAAK K_2P_ could be a new target for the treatment of retinal degeneration–related diseases.

## Abbreviations

AMD: age-related macular degeneration
CNS: central nervous system
Cyt-c: cytochrome c
K_2P_: two-pore domain potassium channels
PR: photoreceptor
Riluzole: 2-amino-6-trifluoromethoxy benzothiazole
RPE: retinal pigment epithelial
RT: room temperature
t-BHP: tert-butyl hydroperoxide
TRAAK: weakly inward rectifying potassium channels
*Δψm*: mitochondrial action potential
